# Early Postoperative Intra-Abdominal Pressure and Heart Rate in Relation to Time to Trophic Enteral Feeding After Posterolateral Congenital Diaphragmatic Hernia Repair: An Exploratory Cohort Study

**DOI:** 10.3390/children13081099

**Published:** 2026-08-18

**Authors:** Raluca-Alina Gogolan, Catalin-Gabriel Cirstoveanu, Ana-Mihaela Bizubac, Mariana-Carmen Heriseanu, Carmina Nedelcu, Adrian-Iustin Georgevici, Nicolae Sebastian Ionescu

**Affiliations:** 1 Doctoral School, “Carol Davila” University of Medicine and Pharmacy, 050474 Bucharest, Romania; raluca-alina.mocanu@drd.umfcd.ro (R.-A.G.); mariana-carmen.iliescu@drd.umfcd.ro (M.-C.H.); carmina.georgescu@drd.umfcd.ro (C.N.); 2 Department of Pediatric Surgery, “Maria Sklodowska Curie” Emergency Clinical Hospital for Children, 077120 Bucharest, Romania; sebastian.ionescu@umfcd.ro; 3 Neonatal Intensive Care Unit, “Maria Sklodowska Curie” Emergency Clinical Hospital for Children, 077120 Bucharest, Romania; ana-mihaela.bizubac@umfcd.ro; 4 Department of Neonatal Intensive Care, “Carol Davila” University of Medicine and Pharmacy, 050474 Bucharest, Romania; 5 Department of Anesthesiology, St. Josef Hospital of Ruhr-University of Bochum, 44791 Bochum, Germany; 6 Department of Pediatric Surgery and Orthopedics, “Carol Davila” University of Medicine and Pharmacy, 050474 Bucharest, Romania; 7 Romanian Academy of Medical Sciences, 030171 Bucharest, Romania; 8 Romanian Academy of Scientists, 050044 Bucharest, Romania

**Keywords:** congenital diaphragmatic hernia, enteral feeding, intra-abdominal pressure, abdominal compartment syndrome, postoperative outcomes

## Abstract

**Background:** Advances in neonatal intensive care have improved survival in congenital diaphragmatic hernia (CDH), shifting the clinical focus toward long-term complications such as feeding difficulties. Initiation of enteral feeding varies across centers, with evidence suggesting that intra-abdominal pressure (IAP) may influence time to feeding and clinical decision-making. We explored associations of early postoperative heart rate (HR), recorded Apgar score, and intra-abdominal pressure (IAP) with time to enteral trophic feeding. **Methods**: We retrospectively evaluated 28 consecutive neonates undergoing CDH repair at a national referral center between January 2022 and June 2025. Trophic enteral feeding (≥10 mL/kg/day for ≥24 h) was the primary endpoint. Aalen–Johansen estimates and two parsimonious Fine–Gray models (HR plus Apgar; IAP plus Apgar) were primary analyses and cause-specific Cox models were sensitivity analyses. **Results:** Within 30 postoperative days, trophic enteral feeding was achieved in 21 cases, six patients died before feeding, and one did not achieve feeding before censoring. Each 10 bpm higher HR was associated with lower feeding incidence (subdistribution HR 0.62, 0.46–0.85; *p* = 0.003); the cause-specific point estimate was also below 1 (HR 0.65, 0.47–0.89; *p* = 0.007). Mean IAP was not clearly associated with feeding incidence (subdistribution HR 1.01 per mmHg, 0.82–1.25; *p* = 0.925). Both Cox models failed global proportional-hazards tests. **Conclusions**: Each 10 bpm increase in HR was associated with a longer time to trophic enteral feeding, and the cause-specific sensitivity analysis pointed in the same direction. Because both estimates derive from the same 28 infants, their agreement is expected rather than corroborative. These findings are exploratory and do not establish causality, individual predictive value, or superiority of HR over IAP. Prospective multicenter studies with adequate sample sizes are needed to confirm these findings.

## 1. Introduction

Congenital diaphragmatic hernia (CDH) is a complex and potentially life-threatening condition characterized by a congenital defect in the diaphragm leading to herniation of abdominal organs into the thoracic cavity [1]. CDH survival has significantly improved in recent years, approaching 80%, due to advances in neonatal intensive care [2,3,4]. Nevertheless, increased survival has highlighted postoperative feeding difficulties among survivors, who often require prolonged intensive care [1,5,6]. Successful transition from parenteral to enteral nutrition is associated with reduced rates of catheter-associated sepsis, enterocolitis, parenteral nutrition-associated liver disease and growth failure; however, the timing of initiation of postoperative feeding is highly variable across centers [6,7,8,9,10].

The mismatch between the volume of previously herniated viscera in the thoracic cavity and the volume of the abdominal cavity could lead to abdominal compartment syndrome (ACS) after CDH surgical repair and may require delayed abdominal fascial closure (DFC). A literature review focusing on DFC requirement or ACS development after CDH repair revealed 112 cases of DFC and six cases of ACS among the 16 studies included in the analysis [11]. A five-year study of Canadian Association of Pediatric Surgery Network database highlighted 12% DFC requirement in patients with CDH, and delayed resumption of enteral nutrition was observed in patients who required DFC or developed ACS [12]. Correlations between intra-abdominal pressure (IAP) and time to achieve full enteral feeding have resulted in recommendations for guiding enteral feeding based on IAP measurement [13].

Nevertheless. consideration should be given to the fact that the thresholds for intra-abdominal hypertension (IAH) differ from those established for adults. The World Society of the Abdominal Compartment Syndrome (WSACS) defines intra-abdominal hypertension in children as an elevation in IAP > 10 mmHg and ACS as a persistent IAP elevation >10 mmHg associated with a new organ dysfunction or exacerbating a pre-existing dysfunction [14]. However, published surveys highlight persistent uncertainty regarding diagnostic cut-offs of IAH and ACS in children [15,16,17].

Cardiovascular parameters provide a complementary perspective regarding feeding tolerance in neonates. Postoperative tachycardia reflects residual pulmonary hypertension, systemic inflammation and inadequate cardiac output, factors which are known to impair gut perfusion and feeding tolerance [4,18]. Predictive studies regarding heart rate (HR) characteristics have linked cardiac rhythm disturbances to adverse neonatal outcomes, suggesting the heart may integrate multisystem stressors effectively [19,20].

We therefore conducted an exploratory pilot-scale analysis to examine the relationship between postoperative IAP, HR, and time to achieve trophic enteral feeding after posterolateral CDH repair.

## 2. Materials and Methods

### 2.1. Study Design

We performed a retrospective cohort study at the Maria Sklodowska Curie Emergency Clinical Hospital for Children, Bucharest, Romania, a national referral center, including consecutive neonates who underwent posterolateral CDH repair between 1 January 2022 and 30 June 2025.

Inclusion criteria required a surgically confirmed posterolateral CDH, operative repair within the first postnatal week, and availability of postoperative monitoring data.

We excluded patients who died prior to surgical intervention or within 24 h postoperatively.

This study is reported in accordance with the Strengthening the Reporting of Observational Studies in Epidemiology (STROBE) guidelines.

Written informed consent was obtained upon admission from the parents of all subjects involved in the study. As our institution is a teaching hospital, the informed consent form includes a section granting permission for participation in clinical teaching and for the use of data in research studies. Additionally, the study was approved by the Institutional Ethics Committee.

### 2.2. Data Collection

Clinical information was extracted from patients’ medical records, operative reports and bedside nursing documentation.

Variables recorded at baseline encompassed sex, gestational age, birth weight, prenatal diagnosis, delivery mode, Apgar scores and defect side. Operative details included time from birth until surgical repair, surgical approach, prosthetic use for diaphragm repair or for abdominal wall closure and extracorporeal membrane oxygenation (ECMO) utilization when applicable. Postoperative data included HR, blood pressure, oxygen saturation, mode of ventilation and the air pressures required, diuresis, intestinal transit, IAP, feeding, death, and clinically recorded ACS.

All data were anonymized, and no personal identifiable information was included.

### 2.3. Exposure Definitions

HR was calculated as the arithmetic mean of routinely charted values on postoperative days 0, 1, and 2. HR was analyzed continuously per 10 bpm increase.

Five-minute Apgar score was assessed by the neonatologist at birth. Mean postoperative HR and the five-minute Apgar score are not overlapping measurements since Apgar is assessed at five minutes of life. However, they are expected to index the same underlying illness severity and joint estimates were interpreted cautiously.

Postoperative IAP was measured during the first three postoperative days and is described in detail in Section 2.4. For analysis, IAP was summarized as the arithmetic mean of the transvesical values recorded on postoperative days 0, 1, and 2, mirroring the HR exposure window, and was analyzed continuously per 1 mmHg increase.

### 2.4. IAP Monitoring

We followed WSACS recommendations for children regarding IAP measurement [14], which requires technical considerations as the instillation volume and patient positioning could influence the accuracy of the recorded values [21,22]. We adhered to the same protocol of IAP measurement that we used in our previously published abdominal wall defects series [23]: intravesical pressure was recorded via an indwelling urinary catheter connected to a pressure transducer. The Foley catheter was connected to a three-way stopcock and one of the ports was attached to the urine collecting bag and the other port to the pressure transducer that was subsequently linked to the multiparameter monitor. During pressure measurement, the patient was positioned supine, with no elevation of the head of the bed, and the transducer was zeroed at the mid-axillary line. To perform the measurement, 1 mL/kg (minimum 3 mL and maximum 25 mL) room-temperature sterile NaCl 0.9% solution was instilled in the bladder via the transducer flush port. Pressures were documented immediately post-repair in the operating room, upon arrival to the Neonatal Intensive Care Unit, one hour after surgery and at least once daily until postoperative day three.

### 2.5. Outcomes

The primary endpoint was time from surgical repair to initiation of trophic enteral feeding, defined as ≥10 mL/kg/day for ≥24 consecutive hours without subsequent cessation. This volume was chosen as an early postoperative marker of enteral tolerance and intestinal adaptation. The threshold value of ≥10 mL/kg/day for trophic enteral feeding was selected based on previously published studies regarding the postoperative nutritional management of neonates [24,25,26]. Infants alive without feeding or death at postoperative day 30 were administratively censored. This early endpoint is not equivalent to full enteral feeding. Death before feeding precluded the feeding event and was treated as a competing event.

Secondary endpoints included decompressive laparotomy or delayed abdominal closure and 30-day all-cause mortality. Compartment syndrome was defined clinically as progressive abdominal distension associating or further impairing at least one organ dysfunction (respiratory, renal, or hemodynamic), prompting decompression and an IAP measuring >10 mmHg before decompression.

### 2.6. Missing Data Handling

HR, Apgar, and IAP were complete for all 28 landmark participants. Recorded feeding times were complete for all 21 infants who achieved the endpoint. Six deaths occurred before feeding within the 30-day window and were analyzed as competing events. A seventh infant was alive and unfed at day 30 and was administratively censored there. This patient died on postoperative day 47, outside the analysis window. The death is a censored observation rather than a competing event. No analytic variable was imputed, and both models used the same complete population.

### 2.7. Statistical Analysis

#### 2.7.1. Descriptive and Competing-Risk Analysis

Continuous variables were summarized by median, interquartile range, and categorical variables by counts and percentages. Baseline characteristics are presented for the full repaired cohort without significance tests between data-derived groups. For the day-3 landmark population, cumulative incidence of sustained trophic feeding and death before feeding was estimated by the Aalen–Johansen method. Death was not treated as non-informative censoring. Follow-up time was measured from the day of surgical repair. The day-3 landmark denotes completion of the exposure window rather than left truncation: no feeding event or death occurred before postoperative day 3. Therefore, every infant was event-free throughout the exposure window by construction, and conditioning on reaching day 3 excluded no one, introducing no immortal time. The two feeding events recorded on day 3 were retained at that time.

#### 2.7.2. Association Models and Sensitivity Analyses

Fine–Gray regression estimated associations with the subdistribution hazard of feeding. To constrain model complexity, two deliberately restricted parsimonious models were fitted: mean postoperative HR per 10 bpm plus Apgar score, and mean postoperative IAP per mmHg plus Apgar score. Both used the same eligibility criteria, exposure window, outcome definition, and complete-case population. These were descriptive association models, not prediction models.

Cause-specific Cox regression was used as a sensitivity analysis with a distinct estimand: the instantaneous feeding rate among infants still alive and unfed. Scaled Schoenfeld-residual tests assessed proportional hazards, and exploratory log-time interactions assessed proportionality in Fine–Gray models. Because both Cox models failed the global proportional-hazards test and Apgar showed term-level time dependence in Fine–Gray diagnostics, constant effect estimates are reported only as compact summaries with explicit caution.

No variable selection, interaction model, subgroup model, data-derived cutoff, dichotomized risk score, receiver operating characteristic analysis, calibration assessment, bootstrap correction, or risk-surface extrapolation was performed. Avoiding post hoc cutpoints and dichotomization was especially important in this small cohort. Estimates are reported with 95% confidence intervals. *p* values are descriptive and no multiplicity adjustment was applied; interpretation emphasizes magnitude, direction, uncertainty, and cross-estimand coherence rather than binary significance.

Analyses were conducted in R version 4.6.0 using survival 3.8-6, cmprsk 2.2-12, and ggplot2 4.0.3. The complete reproducible script generated the cohort audit, estimates, tables, figures, diagnostics, and session information.

## 3. Results

### 3.1. Study Population and Clinical Characteristics

**Figure 1:**
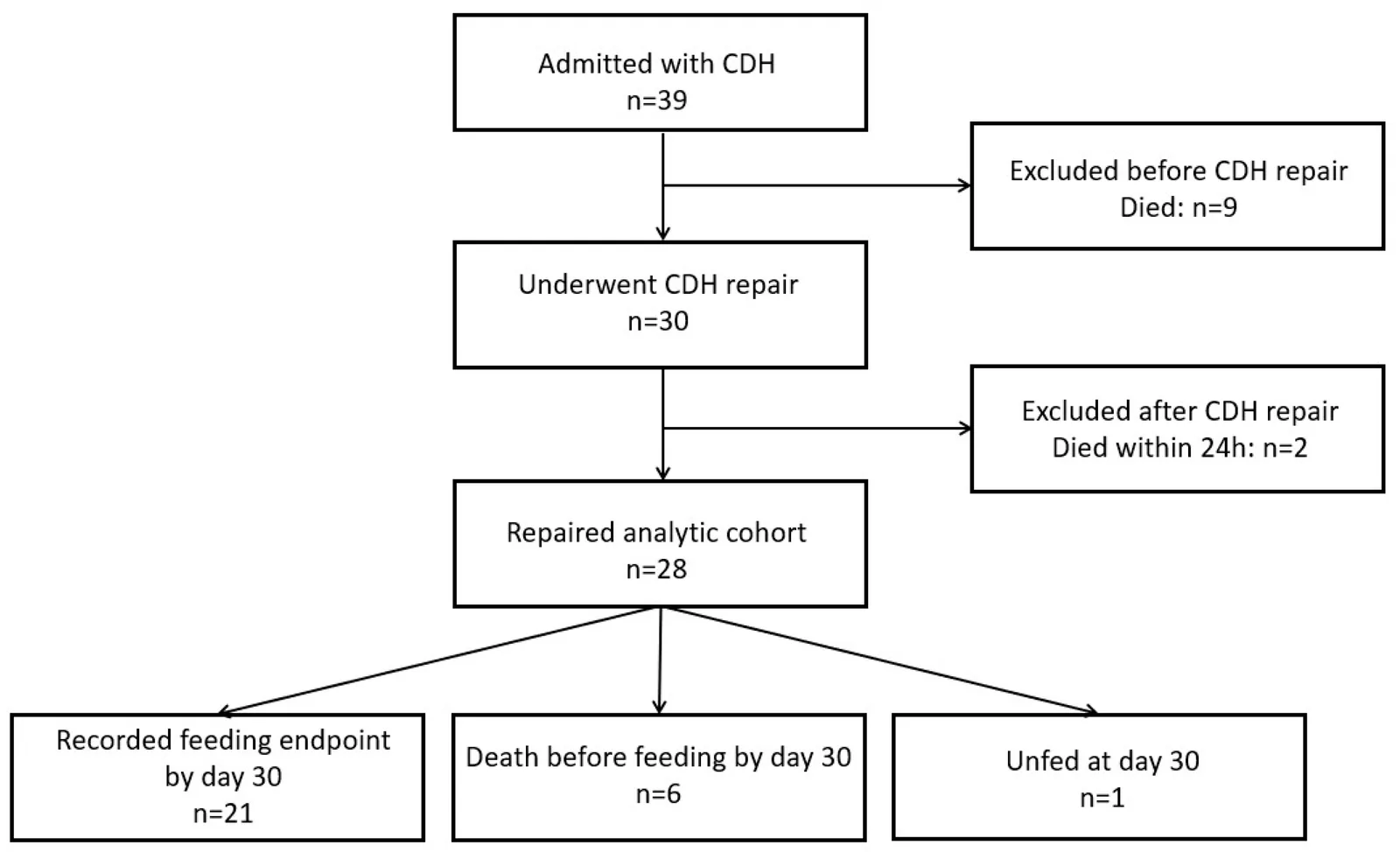
Flowchart of patient selection and study cohort formation.

During the study period, 39 patients with CDH were admitted to the Neonatal Intensive Care Unit. We excluded 11 patients from the study: two neonates with severely compromised status that died within 24 h after surgery, and nine neonates who died before surgery could be performed. Therefore, 28 patients who underwent CDH repair were analyzed (Figure 1).

Among 28 neonates, 22 were delivered via cesarean section, and six via vaginal delivery. Prenatal imaging confirmed CDH in 25 of 28 cases. The cohort included 17 males and 11 females. Median gestational age was 38 weeks and the median birth weight 3080 g. According to the Lally classification [27], two patients were classified as type A CDH, 11 as type B, 13 as type C, and two as type D. Five patients had right-sided diaphragmatic defect and 23 had left-sided diaphragmatic defect. Concerning the surgical technique, thoracoscopic approach was employed in four cases, and in 24 cases, open transabdominal repair of the diaphragm was performed. Patch repair of the diaphragm using a dual-surface expanded polytetrafluoroethylene mesh was required in nine cases. One patient required DFC due to observed intraoperative abdominal wall tension that precluded primary closure, and a dual-surface expanded polytetrafluoroethylene mesh was used for abdominal wall closure. Three patients underwent ECMO. Seven patients died before feeding could be initiated, six of them within 30 postoperative days and one of them on postoperative day 47. Two infants (7.1%) developed postoperative ACS and required abdominal decompression and Silobag placement or Schuster procedure with sterile urine bag (Table 1).

### 3.2. Time to Achieve Trophic Enteral Feeding and Death Before Feeding

By postoperative day 30, 21 infants (75%) achieved trophic enteral feeding, six (21.4%) died before feeding, and one (3.6%) was alive but did not achieve enteral feeding. Among the 21 infants with a feeding event, the recorded postoperative day median was 7 (IQR 5–14; range 3–30).

**Figure 2:**
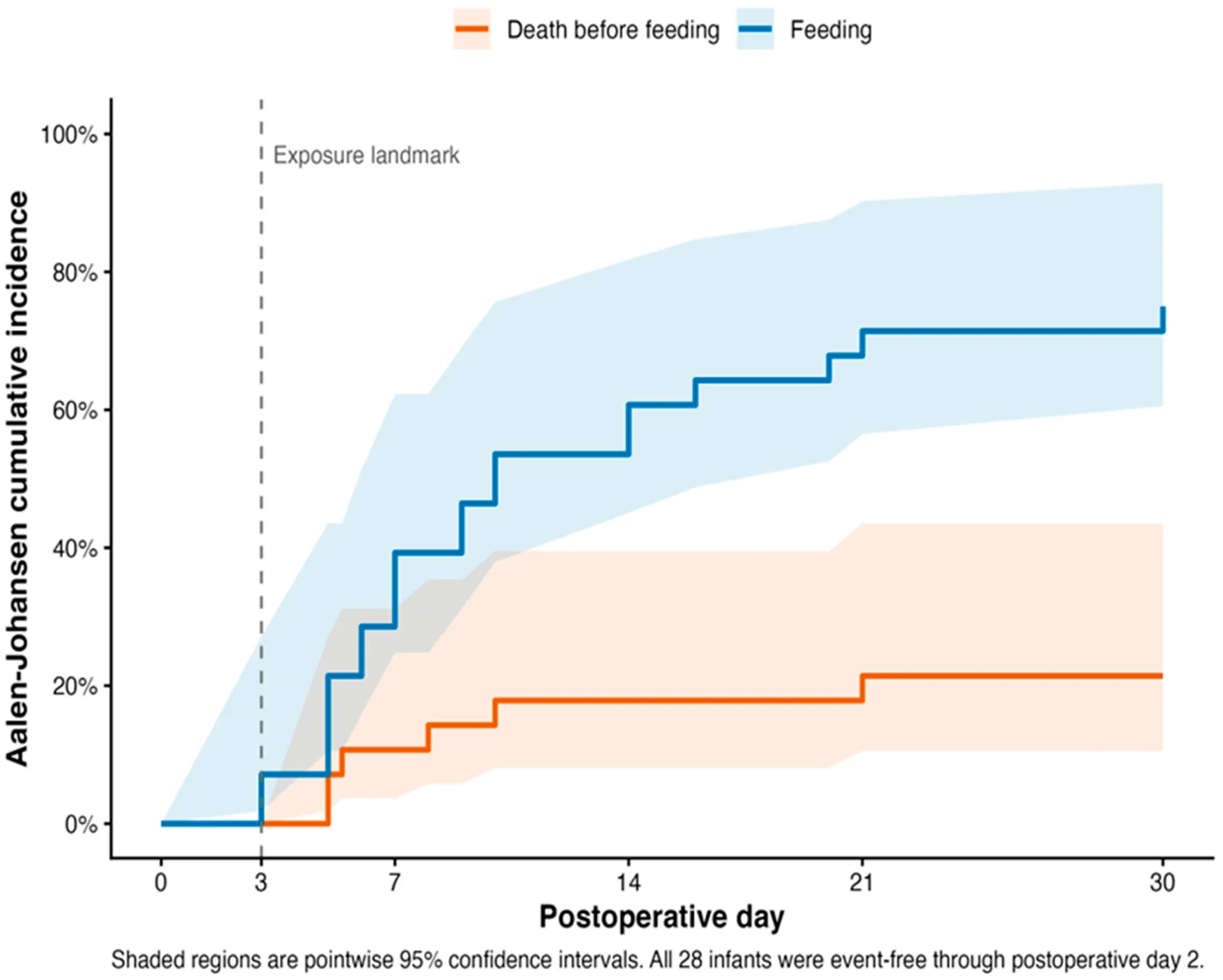
Aalen–Johansen cumulative incidence of the recorded feeding endpoint and death before feeding through postoperative day 30, with pointwise 95% confidence intervals. HR and IAP were summarized on postoperative days 0 to 2. Two feeding events were recorded on day 3, but within-day timestamps were unavailable.

Feeding cumulative incidence was 39.3% (95% CI 24.8–62.3%) at day 7, 60.7% (45.1–81.8%) at day 14, and 75.0% (60.6–92.9%) at day 30. The corresponding death-before-feeding incidence was 10.7% (3.7–31.2%), 17.9% (8.1–39.5%), and 21.4% (10.5–43.6%), respectively (Figure 2). These confidence intervals are wide and reflect sparse-data imprecision in a cohort of 28 infants, therefore the point estimates should not be read as precise.

### 3.3. Factors Associated with Time to Trophic Enteral Feeding

In the Fine–Gray model containing mean postoperative HR and recorded Apgar score, each 10 bmp increase in HR was associated with a lower probability of achieving trophic enteral feeding (shR 0.62, 95% CI 0.46–0.85, *p* = 0.003). The Apgar shR was 1.31 per point (0.74–2.32; *p* = 0.355).

In the cause-specific sensitivity model, the HR point estimate was also below 1 (cause-specific HR 0.65 per 10 bpm, 95% CI 0.47–0.89; *p* = 0.007). The Apgar estimate was 1.28 per point (0.82–2.00; *p* = 0.275). The HR + Apgar Cox model failed the global proportional-hazards test (*p* = 0.026), driven by detected time dependence for Apgar (*p* = 0.008; HR term *p* = 0.949). Therefore, these hazard ratios should be interpreted as average effect over the follow-up period, rather than a constant effect.

In the Fine–Gray IAP + Apgar model, mean IAP was not clearly associated with feeding (shR 1.01 per mmHg, 95% CI 0.82–1.25; *p* = 0.925). The Apgar shR was 1.68 (0.96–2.92; *p* = 0.068). Cause-specific estimates were 1.27 per mmHg for IAP (0.999–1.62; *p* = 0.050) and 1.79 per point for Apgar (1.14–2.79; *p* = 0.011). The cause-specific IAP interval includes 1, so this estimate is not distinguishable from the null, and the IAP + Apgar Cox model failed the global proportional-hazards test (*p* = 0.001) with detected time dependence for both terms. In a prespecified sensitivity analysis summarizing IAP over the same window by its maximum rather than its mean, the two summaries correlated at r = 0.93 and the Fine–Gray estimate was materially unchanged (shR 1.02 per mmHg, 95% CI 0.84–1.23; *p* = 0.854).

Exploratory Fine–Gray joint log-time tests did not reject proportionality for HR + Apgar (*p* = 0.116) or IAP + Apgar (*p* = 0.110), although Apgar term-level interactions were detected in both models (*p* = 0.040 and *p* = 0.042). Term-level tests did not detect evidence of time dependence for HR (*p* = 0.239) or IAP (*p* = 0.504), although the sparse cohort limits the sensitivity of these diagnostics. The proportionality failures were therefore driven by Apgar rather than by the exposures: the HR term satisfied proportionality in both frameworks (cause-specific *p* = 0.949; Fine–Gray *p* = 0.239). Because Apgar is the sole adjustment covariate, its time dependence weakens the adjusted interpretation, and the direction of the HR association is the more defensible element of these models.

**Figure 3:**
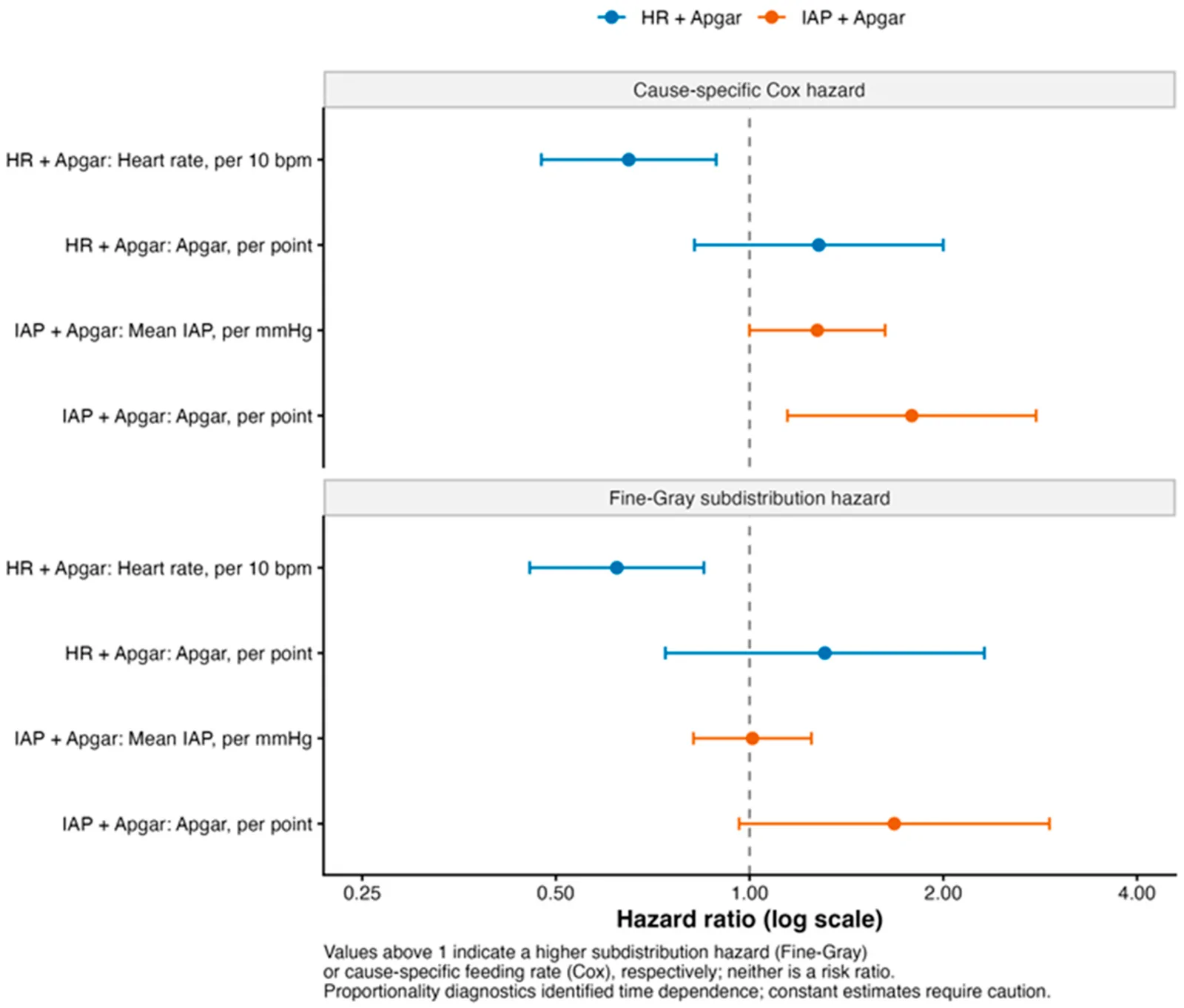
Cause-specific sensitivity model and Fine–Gray model. Fine–Gray subdistribution hazard ratios relate covariates to the cumulative incidence of feeding in the presence of competing death. Cause-specific hazard ratios relate covariates to the instantaneous feeding rate among infants alive and unfed.

The results of the cause-specific Cox and Fine–Gray models are summarized in Figure 3.

Table 2 summarizes the results of the two models used to assess factors associated with time to trophic enteral feeding. Interpretation of the cause-specific estimates must account for the proportional-hazards violations described above.

### 3.4. Abdominal Compartment Syndrome

Two infants (7.1%) developed clinical abdominal compartment syndrome confirmed by IAP measurement, requiring emergency decompressive procedures: one patient underwent a Schuster procedure using a sterile urine bag sutured to the abdominal wall, whereas the other underwent Silobag placement. These cases occurred despite intra-abdominal pressures of only 8 and 11 mmHg at the time of abdominal wall closure and immediately postoperatively.

## 4. Discussion

### 4.1. Principal Findings

In this small retrospective cohort, each 10 bpm higher mean HR over the first three postoperative days was associated with longer time to achieve trophic enteral feeding in the Fine–Gray model. The HR point estimate was also below 1 in the cause-specific sensitivity analysis. Mean IAP was not clearly associated with feeding incidence. Apgar estimates varied between models and the assessment of time-varying effects suggested its effect was not constant over follow-up.

The HR point estimate was below 1 in the primary Fine–Gray model and in the cause-specific sensitivity analysis, an internally consistent direction within this dataset. Because both estimates arose from the same small cohort and the cause-specific model failed the global proportional-hazards test, this pattern is useful only for hypothesis refinement. It is not confirmation, replication, robustness, model stability, or proof. HR, Apgar, and IAP can all reflect global illness severity, treatment, and clinician behavior. The analysis therefore does not support individualized probabilities, a bedside score, a causal effect, or superiority of HR over IAP.

### 4.2. Interpretation of HR and Apgar Score

Heart rate integrates cardiac performance [4,18], inflammatory burden, and metabolic stress, and prior neonatal studies link heart-rate variability with adverse outcomes [19,28]. Although studies correlate HR characteristics with illness severity scores that include feeding intolerance as a component, these reports do not validate HR as a marker of feeding readiness [20].

Apgar score is a well-established assessment method of neonatal condition, and evidence demonstrated association between lower Apgar score (values below six) and adverse neonatal outcomes [29].

Joint HR and Apgar coefficients cannot be interpreted as separate mechanisms because the measures overlap and may reflect the same underlying illness severity. Detected Apgar time dependence and wide confidence intervals further limit independent interpretation.

### 4.3. IAP and Comparison with Prior Feeding Studies

Consensus guidelines for nutrition in CDH patients exist [9], but the optimal timing of enteral feeding following CDH repair remains unclear, as highlighted by several studies [8,30,31]. Building on Williams’ paper [32], which recommends trophic feedings, a multicentric prospective study on newborn surgical patients demonstrated that early enteral nutrition may promote earlier stool evacuation and reduces the time required to achieve full enteral feeding [33].

In a ten-year study regarding enteral feeding in infants with CDH, Zozaya et al. found that patch repair of the diaphragm was associated with a longer time to enteral feeding [6]. In our study, among the nine patients who required patch repair of the diaphragm, five neonates died prior to enteral feeding.

Thambusamy and colleagues reported a positive correlation between duration of intra-abdominal hypertension and time to achieve full enteral feeding, recommending IAP monitoring to guide feeding decisions [13]. Our study included only time to achieve trophic enteral feeding as the endpoint; therefore, no conclusions regarding this association can be drawn from this cohort.

The IAP estimates did not provide clear evidence of an association with feeding in this cohort, but the estimates were imprecise and the cause-specific model violated proportional hazards. A null or uncertain estimate in 28 infants is not evidence that IAP lacks clinical value or that HR performs better.

### 4.4. Implications for Clinical Practice

Although IAP monitoring has an important role in evaluating intra-abdominal hypertension and ACS, this study examined only an exploratory association with feeding timing. These results do not justify changing feeding protocols based on IAP or HR thresholds.

The practical contribution is limited to a hypothesis that warrants further investigation. The observed direction of the HR within our cohort may provide the rationale and design of an adequately powered prospective multicenter evaluation with prespecified exposure timing, standardized feeding assessment, complete treatment and severity data, and explicit competing-risk methods. Any later risk model or bedside threshold would require appropriate development methods and independent external validation.

### 4.5. Strengths and Limitations

Strengths include consecutive enrollment at a national referral center, evaluation of HR and IAP in the same repaired cohort and exposure window, no missing Apgar, HR or IAP data, and explicit handling of death as a competing event. Reporting both Fine–Gray and cause-specific models improves transparency regarding the question addressed by each model.

The main limitation is the small, retrospective, single-center cohort. With 21 feeding events and six competing deaths, estimates are vulnerable to sparse-data variation and cannot be considered confirmed, externally validated, or suitable for individual prediction.

Although the Apgar score remains a widely used clinical indicator of neonatal status, the limitation resides in the inter-observer variability and heterogeneity in Apgar score reporting. Assessment of the skin color could be influenced by ethnicity and the intensity and color tone of ambient lighting conditions and interobserver variability were reported in assessing muscle tone and reflex irritability [34]. In this regard, various studies reported considerable variation in assessing Apgar scores [29,35,36].

Considering the complex physiology of neonates with CDH, pulmonary hypertension and vasoactive treatment represent unmeasured confounders influencing heart rate. Uniform description of contralateral lung hypoplasia, malrotation or volvulus, bowel injury, delivery-room ventilation, persistent pulmonary hypertension and associated cardiac anomalies was unavailable.

Intermittent IAP monitoring may constitute a limitation of this study, as two patients developed ACS despite initially lower IAP values, with elevated IAP and ACS signs detected at one and six hours, respectively. This finding suggests that continuous IAP monitoring could improve early detection of increasing in IAP.

The absence of a standardized feeding protocol is also a limitation, although this may represent an important direction for future research and prospective interventional studies.

### 4.6. Future Directions

Prospective multicenter studies should prespecify HR and IAP measurement windows and covariates, and include treatment data. Prediction performance, calibration, clinical utility, and external validation should be evaluated only after a model has been prospectively specified and developed.

## 5. Conclusions

In this exploratory cohort of neonates who underwent CDH repair, each 10 bpm increase in HR was associated with a longer time to trophic enteral feeding in both the Fine–Gray analysis and the cause-specific sensitivity analysis. This consistent direction of effect within the cohort is not confirmation, replication, proof, causality, model validation, individual risk estimation, or evidence that HR is superior to IAP. It does, however, provide a clinically plausible, prospectively testable association that can inform the rationale and design of an adequately powered multicenter evaluation with prespecified measurements, explicit competing-risk methods, and standardized feeding assessment.

## Figures and Tables

**Table 1:** Baseline, operative and outcome characteristics.

Characteristic	Overall (N = 28)
Cesarean delivery	22 (78.6%)
Prenatal diagnosis	25 (89.3%)
Male sex	17 (60.7%)
Preterm birth (<37 weeks)	7 (25%)
Apgar score	7 (5.8–8.0)
Birth weight (g)	3080 (2788–3342)
Left-sided defect	23 (82.1%)
Open transabdominal repair	24 (85.7%)
Thoracoscopic repair	4 (14.3%)
Patch diaphragmatic repair	9 (32.1%)
Delayed fascial closure	1 (3.6%)
Time from birth until repair (h)	30.5 (22.8–48.0)
ECMO	3 (10.7%)
Death before feeding by day 30	6 (21.4%)
Unfed at day 30	1 (3.6%)
Abdominal compartment syndrome	2 (7.1%)

Values are n (%) or median (interquartile range).

**Table 2:** Associations of early postoperative measures with the recorded feeding endpoint in competing-risk and cause-specific analyses.

Model and Variable	Fine–Gray Subdistribution HR (95% CI)	*p* Value	Cause-Specific HR (95%CI)	*p* Value
Model 1: mean HR; per 10 bpm	0.62 (0.46–0.85)	0.003	0.65 (0.47–0.89)	0.007
Model 1: recorded Apgar; per point	1.31 (0.74–2.32)	0.355	1.28 (0.82–2.00)	0.275
Model 2: mean IAP; per mmHg	1.01 (0.82–1.25)	0.925	1.2700 (0.999–1.62)	0.050
Model 2: recorded Apgar; per point	1.68 (0.96–2.92)	0.068	1.79 (1.14–2.79)	0.011

## Data Availability

De-identified data are available from the corresponding author upon reasonable request and institutional approval.

## Abbreviations

The following abbreviations are used in this manuscript:

ACS	Abdominal Compartment Syndrome
CDH	Congenital Diaphragmatic Hernia
DFC	Delayed Fascial Closure
ECMO	Extracorporeal Membrane Oxygenation
HR	Heart Rate
IAH	Intra-abdominal Hypertension
IAP	Intra-abdominal Pressure
STROBE	Strengthening the Reporting of Observational Studies in Epidemiology
WSACS	World Society of the Abdominal Compartment Syndrome
